# DNA Recovery from Forensically Relevant Blow Fly Larvae (Insecta, Diptera, Calliphoridae) Kept in Different Preservative Solutions

**DOI:** 10.1007/s13744-026-01366-x

**Published:** 2026-02-20

**Authors:** José Lucas Dias-Silva, Orianna Tamara, Andrés F. Maya-Duque, Eduardo Amat, Luz Miryam Gomez-Piñerez, João Vitor Almeida-Santos, Carina Mara Souza, Tais Madeira-Ott, Aline Marrara Prado, Patricia Jacqueline Thyssen

**Affiliations:** 1 Lab of Integrative Entomology, Dept of Animal Biology, IB, Universidade de Campinas (UNICAMP), Campinas, São Paulo State Brazil; 2 Grupo de Investigación Bioforense, Tecnológico de Antioquia (TdeA) Institución Universitaria, Medellín, Antioquia Colombia

**Keywords:** Blow flies, Techniques, Optimization, Molecular identification, Forensic entomology, Neotropical

## Abstract

There are still many gaps in knowledge about how different preservative solutions can guarantee sufficient DNA recovery for the taxonomic determination of flies of forensic importance. In the present study, we evaluated the recovery and amplification rates of mitochondrial DNA from larvae of the forensically important blow fly *Chrysomya megacephala* (F.) (Diptera, Calliphoridae), preserved for up to 47 days in the solutions commonly used in routine forensic investigations. Some larvae (*N* = 88) were immediately placed in the preservative solution, while another group of larvae (*N* = 88) was killed by immersion in water heated up to 80 °C for 30 s before being preserved at room temperature. In general, it was possible to recover highly pure DNA from larvae subjected to almost all treatments, with yields varying proportionally across different storage intervals. Among the solutions that yielded the highest DNA and consequently achieved successful amplification rates were 99.3% ethanol (> 100 ng/µL, with rates of 95%) and 70% ethanol (20–100 ng/µL, with rates of 90%), primarily up to the 28-day storage interval. The worst DNA yields and low amplification rates (approximately 50%) were associated with the 47-day storage interval, despite the preservative method used, except for Kahle’s solution, which presents no amplification at all. Our results suggest that ethanol at concentrations of 70–99.3% is the most efficient preservative for preserving the integrity and usability of forensically important larvae DNA for diagnostic purposes, particularly within a range of up to 28 days.

**Supplementary Information:**

The online version contains supplementary material available at 10.1007/s13744-026-01366-x.

## Introduction

Knowing the period of insect activity (PIA) or time of colonization (TOC) associated with decaying corpses can be useful for estimating the minimum postmortem interval (minPMI) (Amendt et al. 2007). However, it is essential to access information on the development rate of the species that use this resource (e.g., Lord et al. 1986; Goff and Odom 1987; Grassberger et al. 2003; Sukontason et al. 2007; Vanin et al. 2008; Sanford et al. 2014; Vasconcelos et al. 2014; Thyssen et al. 2018). Identifying one or a set of species is one of the initial steps to associate the information provided by insects with the resolution of forensic questions (Thyssen 2010). This task can be laborious due to the restricted number of diagnostic morphological characteristics inherent to the taxon itself, for example, in Sarcophagidae (Carvalho and Mello-Patiu 2008; Souza et al. 2020), or to the life stage in which they are found, for example, the immature stages of blow flies (Greenberg and Szyska 1984; Wells et al. 1999; Thyssen 2010; Szpila et al. 2013; 2014; 2024; Prado et al. 2023).

Characterization of several genes and advances in molecular knowledge have contributed to overcoming the taxonomic impediment among insect species of forensic importance (e.g., Sperling et al. 1994; Wells and Sperling 2001; Wallman et al. 2005; Boehme et al. 2010; Mazzanti et al. 2010; Meiklejohn et al. 2013; Grella et al. 2015; Madeira et al. 2016; Yusseff-Vanegas and Agnarsson 2017; Shang et al. 2019; Grzywacz et al. 2021; Yan et al. 2021; Shao et al. 2023). However, regardless of the type of analysis, the recovery of sufficient quantity and quality of genetic material to ensure a successful result will depend, above all, on the means used to preserve the sample (Linville et al. 2004; Amendt et al. 2007; Brown et al. 2012).

A considerable number of studies have been dedicated to evaluating the effect of the resources that precede (e.g., bathing in boiling water) and preserve both adult and mostly immature insects. There are reports using freezing (from −20 to −80 °C), combined or not with immersion in preservative solutions such as 70–100% ethanol, 99.7% isopropyl alcohol, 10% formalin, KAA (kerosene, glacial acetic acid), XA (xylene and ethanol), Kahle’s or Pampel’s fluid (formalin, glacial acetic acid, and ethanol), and San Veino (a commercial product containing formaldehyde, phenol, methanol, and synthetic camphor oil) concerning morphology, morphometry (Lord and Burger 1983; Tantawi and Greenberg 1993; Adams and Hall 2003; Day and Wallman 2008; Bugelli et al. 2017; Niederegger 2021; López-García et al. 2024), and, less often, molecular aspects (Brown et al. 2012; Smith 2024).

Although guidelines available in the literature (Amendt et al. 2007) encourage standardization regarding collecting and preserving insect samples from crime scenes, this may be unfeasible in practice. Inaccessibility to the components of such technique (e.g., heated water to prevent deformity of fly larval body segments) or to particular chemical product and distance between the crime scene and the police laboratory, where the samples will be processed, represent some of the obstacles that negatively contribute to this lack of standardization or optimization (Thyssen et al. 2018; Day and Wallman 2008).

After thorough case review, Day and Wallman (2008) reported that ethanol is one of the main solutions used to preserve insects’ immature stages of forensic importance. The cost, ease of obtaining, low toxicity, and satisfactory results obtained from procedures such as molecular (Madeira et al. 2016) or toxicological analysis (Souza et al. 2013) contribute to the choice of ethanol as the best preservative. In controlled experiments, killing larvae in boiling water followed by fixation in ethanol has also been the most commonly recommended (Catts and Haskell 1990; Adams and Hall 2003; Day and Wallman 2008). We aim to evaluate methods for preserving blow fly larvae of forensic interest targeting to access DNA sequences for taxonomic determination, a crucial step within the workflow in forensic entomology practice.

## Material and Methods

### Obtaining Specimens for Study

Colonies of *Chrysomya megacephala* (Fabricius) (Insecta, Diptera, Calliphoridae) were established in the laboratory from actively collected adult flies (22°54′21″S: 47°03′39″W) using bovine liver previously putrefied for 48 h as bait. In the laboratory, 12 couples of this species were anesthetized by low temperature (−20 °C for 3 min), identified (Grella et al. 2015), transferred to screened plastic cages, and kept in a room under controlled conditions (27 ± 1 °C, 70 ± 10% R.H., 12 h photophase). Adult specimens were fed with water and sugar ad libitum. A 50 g portion of fresh raw ground beef was offered to stimulate oviposition. As part of the standardized laboratory routine, the obtained eggs were counted (by weight estimation, using an analytical balance, where 29.5 mg equals 250 eggs), transferred to vials containing fresh ground beef at a ratio of 1 g/egg, and kept in a climate chamber under the same controlled conditions as described for adults.

### Experimental Design and Data Analyses

Third instar larvae (*N* = 176) were removed from the feed 84 h after hatching and previously washed in distilled water to remove residues adhered to the body. Then, a portion of the larvae (*N* = 88) were killed in heated water at 80 °C for 30 s (Adams and Hall 2003). Subsequently, all larvae (*N* = 176) were placed in groups (at least 3 specimens per tube) in different types of preservative solution: 70% ethanol, 99.3% ethanol, 99.7% isopropyl alcohol, and Kahle’s solution (Table 1).

**Table 1.**
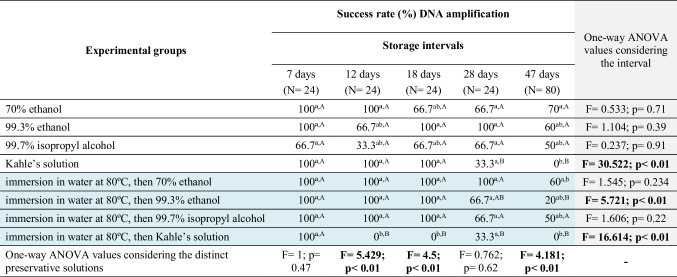
Amplification rate (%) taking into account the experimental groups and storage intervals to which the blow fly larvae *Chrysomya megacephala* were subjected for preservation purposes

Note: Composition of 100 mL of Kahle’s solution: 30 mL of 95% ethanol, 12 mL of formaldehyde, 4 mL of glacial acetic acid, and 60 mL of distilled water. **Bold values** indicate significant differences when *p* < 0.05. Within each column and row, respectively, equal lowercase and uppercase letters are not significantly different

Between 7 and 47 days after preservation, DNA extraction and amplification of a fragment of approximately 600 bp of the cytochrome oxidase I (*CO*I) portion of mitochondrial DNA (mtDNA) was processed using the universal primers LCO1490 and HCO2198 (Folmer et al. 1994). DNA was extracted from the median part of the larval body—between the 4th and 9th segments, which corresponded to approximately 20 mg of mass—using the commercial kit DNeasy Blood and Tissue™ (Qiagen, Valencia, CA, USA). The remaining parts of the larvae—intact anterior and posterior portions, which included the anterior and posterior respiratory spiracles as well as the cephaloskeleton—were stored individually as vouchers and deposited in the entomological collections of TdeA (CETdeA) and LEI (CELEI 2025). A NanoDrop™ 8000 Microvolume UV-Vis spectrophotometer (Thermo Fisher Scientific) and a Qubit 3 fluorometer (Thermo Fisher Scientific) were used to measure the DNA quantity (in ng/µL). The corresponding elution buffer was used as the blank. Optionally, DNA purity in the NanoDrop™ was assessed by calculating the absorbance ratio at 260/280 nm, considering that values ​​between 1.8 and 2.2 indicate greater purity (Glasel 1995; Hassan et al. 2015), while values ​​ ≤ 1.6 indicate the presence of potential contaminants, including nonspecific proteins, among others (Lucena-Aguilar et al. 2016).

Amplifications were done in 25 µL reaction final volume, containing 1× PCR buffer, 0.2 mM of dNTPs mix, 2.5 mM of MgCl_2_, 0.4 µM of each primer, 1 U of Promega GoTaq™ Flexi DNA polymerase, 2.5 µL (30–50 ng) of DNA, and ultra-pure water to complete the volume. The reaction cycle consisted of an initial step of 3 min at 95 °C, followed by 35 cycles of 30 s at 95 °C, 1 min at 50 °C, and 1 min at 72 °C. The last cycle included an extended elongation step of 5 min at 72 °C. PCR amplicons were separated by electrophoresis on a 2% agarose gel in 1× TAE buffer, stained with GelRed™, and visualized under UV light. HyperLadder 1 kb LAB MARK Ladder (Bioline) was used to estimate the size of amplicons.

To assess the usability of the DNA sequences for taxon identity recovery, amplicons from 1–2 specimens from each treatment were purified using EnzSAP™ PCR Clean-Up reagent kit, following manufacturer’s protocols. Subsequently, amplicon sequencing was performed using the Sanger method. Obtained nucleotide sequences were verified and edited using the Geneious Prime™ 2025.0.1. After that, each sequence was used to search homologous *CO*I sequences in the GenBank database, using a BLASTn. Some homologous *CO*I sequences from neotropical *C. megacephala* specimens available at GenBank database were included in our analysis for comparative purposes. Neighbor-Joining (NJ) using the K2-p substitution model and genetic distance analyses were performed using the MEGA7™ software (Kumar et al. 2016), based on the DNA barcoding method (Hebert et al. 2003). The evolutionary model that best represented the dataset was tested using the jModelTest 2™ program (Darriba et al. 2012). For further details on the sequences analyzed in this study, see Supplementary Material 1.

The Shapiro-Wilk test was previously applied to assess whether the data had a normal distribution. One-way ANOVA was performed to evaluate the DNA yield and, when applicable, the degree of DNA purity obtained from larvae exposed under different experimental conditions, using the DNA quantification or the absorbance ratio at 260/280 nm values as response variables. Additionally, the success rate in DNA amplification of larvae subjected to different experimental conditions was evaluated by one-way ANOVA considering either the treatment type or the storage interval. The means were compared using Tukey’s post hoc test. Principal component analysis (PCA) was performed to estimate the correlation between DNA quantification and amplification rates, after standardization (mean = 0, standard deviation = 1). Then, biplots were generated to assess the contributions of variables across the different treatment groups and storage intervals, including confidence ellipses. Values ​​were considered significant when *p* < 0.05. All statistical analyses were performed using R v. 4.4.1 (R Core Team 2024).

## Results

**Fig. 1:**
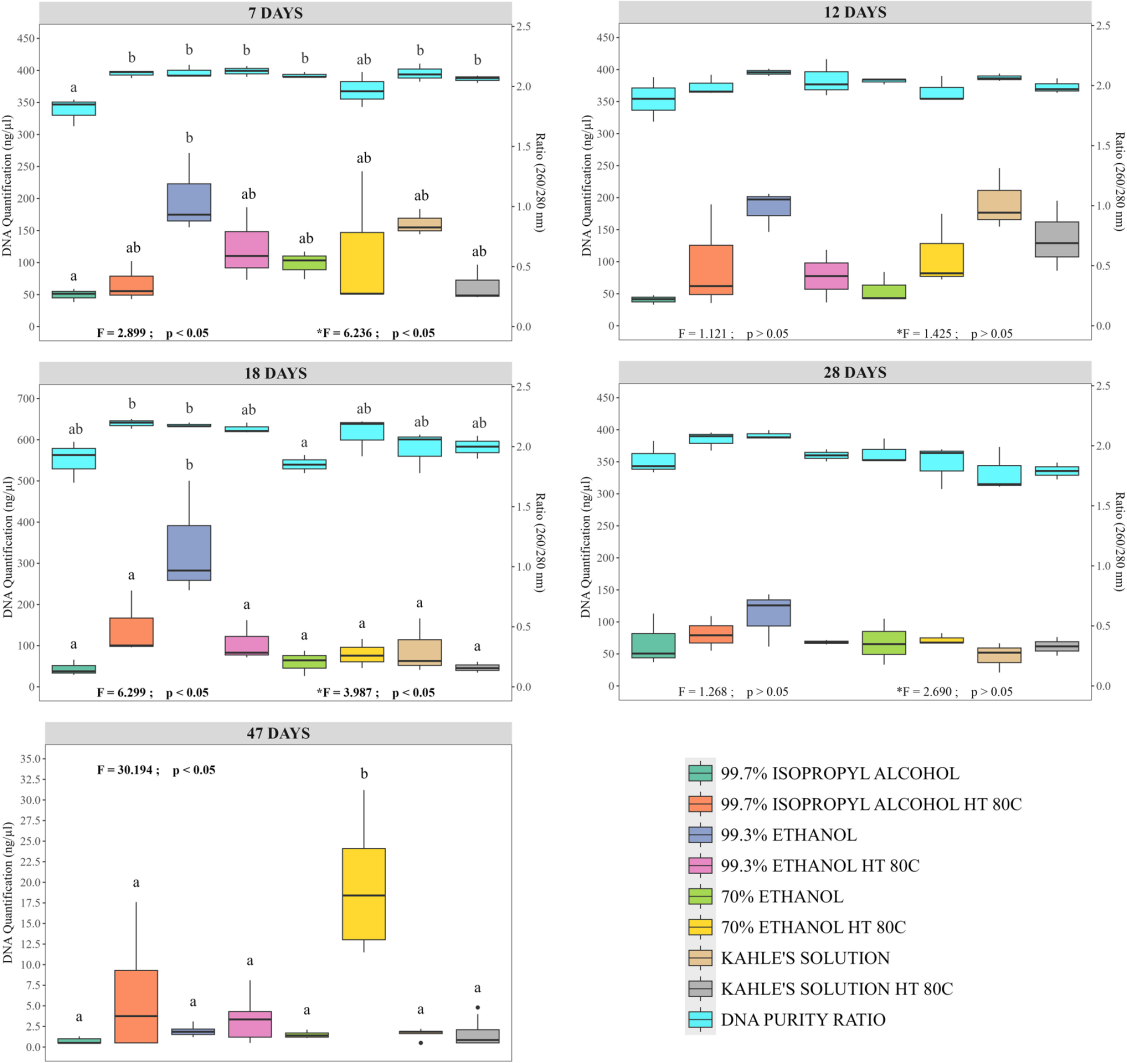
Yield of total extracted DNA (ng/µL) and absorbance ratio (260/280 nm) by experimental groups and storage intervals of *Chrysomya megacephala* larvae evaluated in this study. Note: Bold values indicate significant differences when *p* < 0.05

**Fig. 2:**
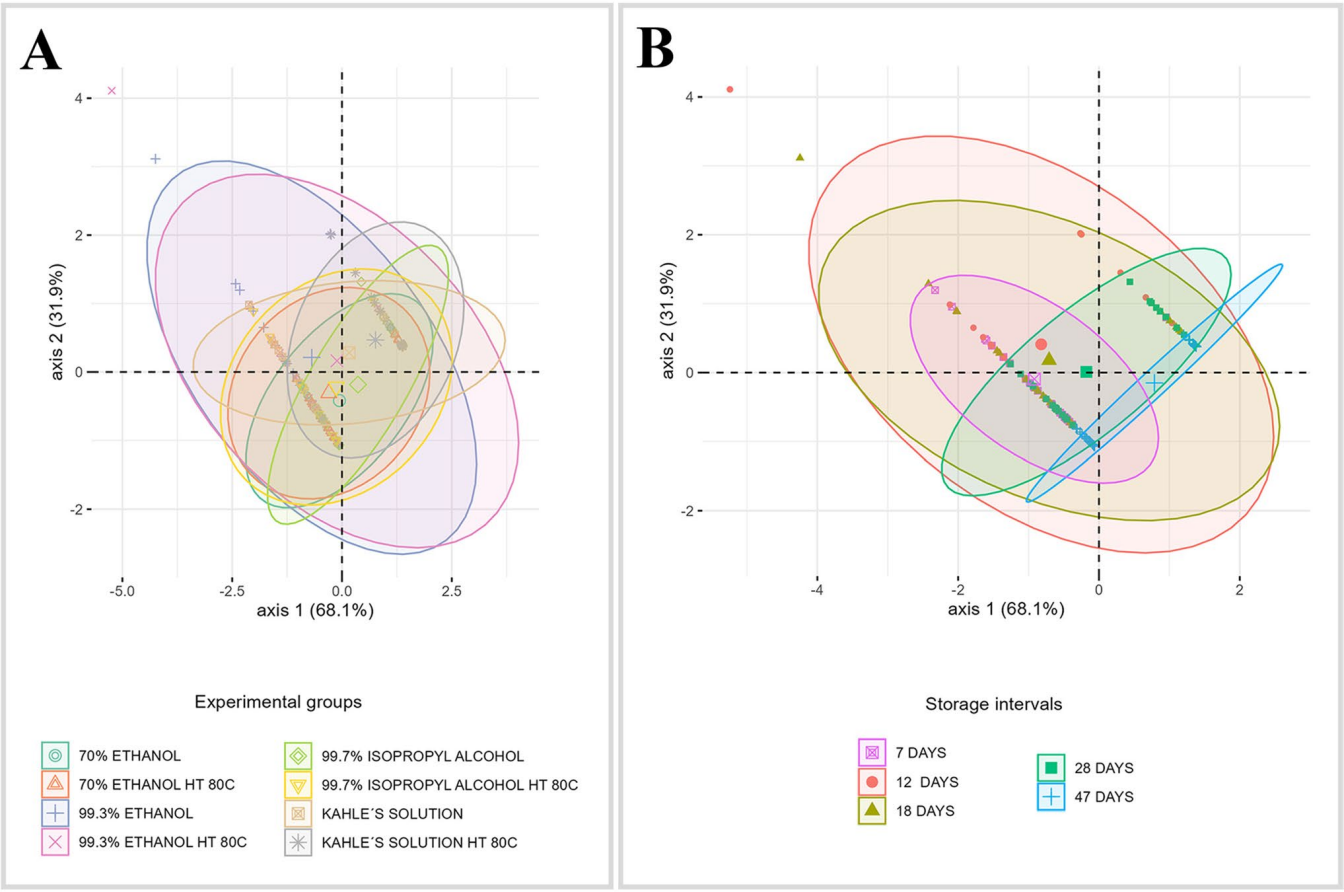
Principal component analysis (PCA) biplot showing the distribution of samples, colored according to experimental groups (**A**) and storage intervals (**B**). The biplot shows the score value and ellipses indicating the within-group variability

In most cases, a considerable yield of total DNA was obtained from *C. megacephala* larvae exposed to different pre-fixation procedures, preservative solutions, and storage intervals (Fig. 1, Supplementary Material 2). DNA concentrations above 100 ng/µL were recovered in 30.8% of the samples evaluated, while for the remaining samples (69.2%), the recovered DNA concentrations were respectively between 20 and 100 ng/µL (50.0%) or below 20 ng/µL (19.2%) (Fig. 1, Supplementary Material 2). Among the preservative solutions that ensure the recovery of a higher DNA yield and consequently successful rates of obtaining amplicons are (i) above 100 ng/µL: 99.3% ethanol (up to 28 days of storage, with a 95% amplification rate) and Kahle’s solution (up to 12 days, with a 77.8% amplification rate) and (ii) 20–100 ng/µL: 70% ethanol (up to 28 days, with a 90% amplification rate) and 99.7% isopropyl alcohol (up to 28 days, with a 72.2% amplification rate) (Figs. 1 and 2, Supplementary Material 2). The worst DNA yields (below 20 ng/µL) were observed after 47 days of storage in any of the preservative solutions evaluated in this study. Although it was possible to obtain an average DNA amplification rate of 50% from samples preserved directly in each of these solutions, 70% ethanol, 99.3% ethanol, and 99.7% isopropyl alcohol, no DNA was amplified when Kahle’s solution was used (Table 1). PCA shows how to select the preservative solution and storage time to obtain better yields and higher DNA amplification rates (Fig. 2).

As expected, DNA concentrations above 20 ng/µL ensured successful rates (79.7%) of amplification of the mtDNA-*CO*I gene (Fig. 2, Supplementary Material 2). Among the 20.3% of larval samples that did not have their DNA amplified are those preserved mainly in Kahle’s solution (47.6%) and 99.7% isopropyl alcohol (28.5%) from a storage interval of 28 days (Fig. 2, Supplementary Material 2). The highest amplification rates were observed among samples stored for up to 7 days in any of the preservative solutions evaluated (Table 1). From the 12th day of storage, significant differences were observed in relation to the type of preservative solution, with the best results obtained between 70 and 99.3% ethanol regardless of whether the larvae were killed directly in the preservative or previously in water at 80 °C (Table 1).

Regarding the degree of purity based on the absorbance ratio at 280/260, 96% of the total DNA recovered was within the parameters that establish a high degree of purity (Fig. 1). Although 4% of the total DNA obtained fell within the absorbance ratio range of 1.6–1.7, particularly associated with samples preserved in 99.7% isopropyl alcohol or Kahle’s solution, it was still possible to establish that the measured DNA was pure, i.e., free of contaminants (Supplementary Material 2).

**Fig. 3:**
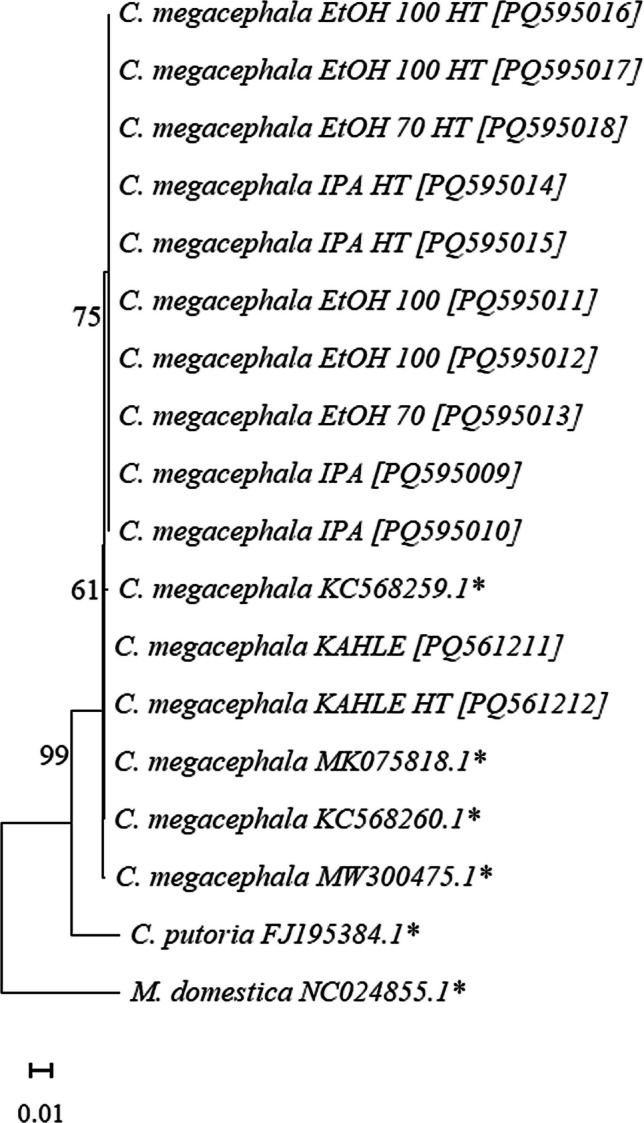
Neighbor-Joining (NJ) tree inferred using a partial ≅ 600 bp *CO*I gene dataset, and following the K2-p substitution model of *Chrysomya megacephala* samples evaluated in this study. Numbers above the branches refer to node supports (bootstrap proportions among 1,000 replicates). *Sequences retrieved from public databases, including that used in this study analysis as outgroup such as *Chrysomya putoria* and *Musca domestica.*

The searches performed in GenBank with the sequences of approximately 600 bp of mtDNA-*CO*I obtained in this study (Supplementary Material 1) returned the expected identification for the species. In the NJ tree topology, it can be seen that the cluster composed of *C. megacephala* specimens evaluated in this study, including those retrieved from GenBank, presented 99% bootstrap support (Fig. 3).

## Discussion

Ethanol with concentrations ranging from 70 to 99.3% has proven to be an efficient resource for preserving biological samples, including other advantages such as low cost and toxicity, easy obtainability, and little or no interference in the processing of samples a posteriori, among others (e.g., Lord and Burguer 1983; Adams and Hall 2003; Day and Wallman 2008; Niederegger et al. 2011; Souza et al. 2013; Madeira et al. 2016). At least one report (Martoni et al. 2019) showed that it was possible to recover DNA from larvae of several Muscidae species kept from 4 to 7 years in absolute ethanol. Sperling et al. (1994) were successful in amplifying DNA from fly larvae kept for 6 months in 75% ethanol. In this study, we demonstrated that samples preserved in ethanol can ensure good yield and allow DNA usability for identification purposes, within a storage period of up to 47 days.

Studies with flies puparium and ants (Brown et al. 2012; King and Porter 2004) have shown successful DNA recovery from samples preserved in isopropyl alcohol. However, after a few weeks, Lord and Burger (1983) observed that fly larvae had their tegument darkened and became more brittle at higher concentrations of isopropyl alcohol. To avoid hardening of larval tegument and impairing the evaluation of anatomical characters, the same authors (Lord and Burger 1983) recommended using isopropyl diluted in water 1:1, but this condition would not ensure DNA integrity. The use of diluted isopropyl alcohol was not evaluated in our study, but we noted that the tegument of *C. megacephala* larvae became darkened after 7 days of storage in this preservative solution (Supplementary Material 3). Furthermore, our results indicated that 99.7% isopropyl alcohol was not as efficient as ethanol in obtaining good DNA yield and successful amplification rates, especially from 28 days of preservation.

As expected, good yield and successful DNA amplification rates were not achieved with samples preserved in Kahle’s solution, particularly after 7 days of storage. Formaldehyde, one of the main components of this solution, crosslinks proteins (Krogmann and Holstein 2010), thus becoming larval tissue samples useless for DNA extraction. However, for anatomical and morphological studies, Kahle’s solution can be useful, since this preservative medium prevents fly larvae tissue from darkening or discoloration, particularly in those necrophagous species (Supplementary Material 3) that generally contain large amounts of bacteria and semi-digested putrefactive material in their digestive tract (Adams and Hall 2003). Unfortunately, no information is available to understand whether Kahle’s solution diluted in water (1:1 or 1:2) could contribute to the long-term preservation of morphological characters without compromising DNA integrity.

The step of immersing fly larvae in heated water to prevent morphological structures collapse, especially of the spines distributed along the body and integument, was shown to be compatible with DNA amplification, as reported in this study. The benefits of stretching larvae to ensure a reliable identification based on morphological examination are well documented in the literature (e.g., Adams and Hall 2003; Amendt et al. 2007; Day and Wallman 2008). Given the inaccessibility of heated water during sample collection at a crime scene, Day and Wallman (2008) evaluated and demonstrated that larvae can be successfully stretched after remaining preserved in ethanol for up to 24 h. Therefore, and also based on our findings, we emphasize to maintain the integrity of the sample for an accurate morphological examination as well as ensuring the obtaining of DNA; larvae should be stretched in water at 80 °C for 30 s before immersion in ethanol or at least 24 h after remaining in it.

DNA barcoding has been frequently used to achieve species diagnoses, including those of forensic importance (e.g., Mazzanti et al. 2010; Brown et al. 2012; Meiklejohn et al. 2013; Madeira et al. 2016; Yusseff-Vanegas and Agnarsson 2017; Martoni et al. 2019; Amat et al. 2023). For this reason, our efforts were focused on achieving reliable identification from a standard universal fragment (Hebert et al. 2003). We also demonstrated that the sample preservation methods used in this study, when appropriately combined with storage intervals, ensure the usability of DNA for identification purposes.

Finally, after collecting samples, whether from a crime scene, decaying corpses, contaminated food, or processed products, or from any study conducted in a laboratory under controlled conditions, be clear about the sample’s purpose and the likely storage time. As shown in this study, it is possible to choose one or more media and methods to ensure the storage of forensically important fly larvae in entomological collections, forensic libraries, and other relevant institutions, including those suitable for long-term storage under conditions that allow for access to DNA or morphological characteristics. Thus, we recommend the use of ethanol at concentrations of 70–99.3% for preserving the integrity and usability of forensically important larvae DNA for diagnostic purposes.

## Supplementary Information

Below is the link to the electronic supplementary material.

ESM1 (DOCX 22.5 KB)

ESM2 (DOCX 67.1 KB)

ESM3 (PNG 3.47 MB)

High Resolution Image (34.6 MB)

## Data Availability

The datasets generated and analyzed during the current study are available from the corresponding author on reasonable request.
