# Supplementary material for: DNA Recovery from Forensically Relevant Blow Fly Larvae (Insecta, Diptera, Calliphoridae) Kept in Different Preservative Solutions
Source: Neotrop Entomol. 2026 Feb 20;55(1):14. doi: 10.1007/s13744-026-01366-x (PMC12920309; doi:10.1007/s13744-026-01366-x)
Supplement: Supplementary file 1 — (DOCX 22.5 KB) [file 13744_2026_1366_MOESM1_ESM.docx]

**DNA recovery from forensically relevant blow fly larvae (Insecta, Diptera, Calliphoridae) kept in different preservative solutions.** Dias-Silva et al.

**Supplementary Material 1**. Overall information on specimens and molecular markers investigated in this study.

| **Species** | **Data of label from the analyzed material** | **GenBank sequence accession ID** |
| --- | --- | --- |
| *Chrysomya megacephala* (Fabricius) | Campinas, SP, Brazil. CETdeA catalog #21417 ^1^ | - |
| *C. megacephala* | Campinas, SP, Brazil. CETdeA catalog #21418 ^1^ | - |
| *C. megacephala* | Campinas, SP, Brazil. CETdeA catalog #21422 | PQ595018 ^1^ |
| *C. megacephala* | Campinas, SP, Brazil. CETdeA catalog #21429 | PQ595017 ^1^ |
| *C. megacephala* | Campinas, SP, Brazil. CETdeA catalog #21434 | PQ595016 ^1^ |
| *C. megacephala* | Campinas, SP, Brazil. CETdeA catalog #21437 | PQ595015 ^1^ |
| *C. megacephala* | Campinas, SP, Brazil. CETdeA catalog #21439 | PQ595014 ^1^ |
| *C. megacephala* | Campinas, SP, Brazil. CETdeA catalog #21441 ^1^ | - |
| *C. megacephala* | Campinas, SP, Brazil. CETdeA catalog #21457 | PQ595013 ^1^ |
| *C. megacephala* | Campinas, SP, Brazil. CETdeA catalog #21467 ^1^ | - |
| *C. megacephala* | Campinas, SP, Brazil. CETdeA catalog #21468 ^1^ | - |
| *C. megacephala* | Campinas, SP, Brazil. CETdeA catalog #21469 | PQ595012 ^1^ |
| *C. megacephala* | Campinas, SP, Brazil. CETdeA catalog #21474 | PQ595011 ^1^ |
| *C. megacephala* | Campinas, SP, Brazil. CETdeA catalog #21478 | PQ595010 ^1^ |
| *C. megacephala* | Campinas, SP, Brazil. CETdeA catalog #21480 ^1^ | - |
| *C. megacephala* | Campinas, SP, Brazil. CETdeA catalog #21482 | PQ595009 ^1^ |
| *C. megacephala* | Campinas, SP, Brazil. CETdeA catalog #21419–21421 ^1^ | - |
| *C. megacephala* | Campinas, SP, Brazil. CETdeA catalog #21423–21428 ^1^ | - |
| *C. megacephala* | Campinas, SP, Brazil. CETdeA catalog #21430–21433 ^1^ | - |
| *C. megacephala* | Campinas, SP, Brazil. CETdeA catalog #21435–21436 ^1^ | - |
| *C. megacephala* | Campinas, SP, Brazil. CETdeA catalog #21438 ^1^ | - |
| *C. megacephala* | Campinas, SP, Brazil. CETdeA catalog #21440 ^1^ | - |
| *C. megacephala* | Campinas, SP, Brazil. CETdeA catalog #21442–21456 ^1^ | - |
| *C. megacephala* | Campinas, SP, Brazil. CETdeA catalog #21458–21466 ^1^ | - |
| *C. megacephala* | Campinas, SP, Brazil. CETdeA catalog #21470–21473 ^1^ | - |
| **Species** | **Data of label from the analyzed material** | **GenBank sequence accession ID** |
| *C. megacephala* | Campinas, SP, Brazil. CETdeA catalog #21475–21477 ^1^ | - |
| *C. megacephala* | Campinas, SP, Brazil. CETdeA catalog #21479 ^1^ | - |
| *C. megacephala* | Campinas, SP, Brazil. CETdeA catalog #21481 ^1^ | - |
| *C. megacephala* | Campinas, SP, Brazil. CETdeA catalog #21482–21496 ^1^ | - |
| *C. megacephala* | [3^rd^ instar larva – vouchers from #CELEI-2025-1 to #CELEI-2025-11, *C. megacephala*; Pelotas, RS, Br]; [reared in laboratory between 2024-2025; Dias-Silva, JL. & cols] ^2^ | - |
| *C. megacephala* | [3^rd^ instar larva – voucher #CELEI-2025-12, *C. megacephala*; São Paulo de Olivença, AM, Br]; [reared in laboratory between 2024-2025; Dias-Silva, JL. & cols] | PQ561211 ^2^ |
| *C. megacephala* | [3^rd^ instar larva – vouchers from #CELEI-2025-13 to #CELEI-2025-23, *C. megacephala*; Pelotas, RS, Br]; [reared in laboratory between 2024-2025; Dias-Silva, JL. & cols] ^2^ | - |
| *C. megacephala* | [3^rd^ instar larva – voucher #CELEI-2025-24, *C. megacephala*; Pelotas, RS, Br]; [reared in laboratory between 2024-2025; Dias-Silva, JL. & cols] | PQ561212 ^2^ |
| *C. megacephala* | [3^rd^ instar larva – vouchers from #CELEI-2025-25 to #CELEI-2025-96, *C. megacephala*; Pelotas, RS, Br]; [reared in laboratory between 2024-2025; Dias-Silva, JL. & cols] ^2^ | - |
| *C. megacephala* | - | MW300475.1 ^3^ |
| *C. megacephala* | - | KC568260.1 ^3^ |
| *C. megacephala* | - | MK075818.1 ^3^ |
| *C. megacephala* | - | KC568259.1 ^3^ |
| *Chrysomya putoria* (Wiedemann) | - | FJ195384.1 ^3^ |
| *Musca domestica* L. | - | NC024855.1 ^3^ |

^1^ Data on the collector, method of obtaining the sample, location and date, and preservation methods are available on the GIBF platform at <https://doi.org/10.15472/o1akfx>

^2^ Vouchers deposited in the Entomological Collection of the Laboratory of Integrative Entomology (CELEI). Additional data can be found at <https://sites.google.com/unicamp.br/lei-dba-unicamp/>

^3^ Sequences retrieved from public databases, including those used in this study analysis as outgroup such as *C. putoria* and *M. domestica*.
