# Supplementary material for: DNA Recovery from Forensically Relevant Blow Fly Larvae (Insecta, Diptera, Calliphoridae) Kept in Different Preservative Solutions
Source: Neotrop Entomol. 2026 Feb 20;55(1):14. doi: 10.1007/s13744-026-01366-x (PMC12920309; doi:10.1007/s13744-026-01366-x)
Supplement: Supplementary file 2 — (DOCX 67.1 KB) [file 13744_2026_1366_MOESM2_ESM.docx]

**DNA recovery from forensically relevant blow fly larvae (Insecta, Diptera, Calliphoridae) kept in different preservative solutions.** Dias-Silva et al.

**Supplementary Material 2**. Amount of total DNA recovered based on the body mass of each processed *Chrysomya megacephala* larva, spectrophotometric ratio, and success in obtaining mtDNA-COI amplicons, taking into account the different types of preservative solutions and sample storage interval.

| **Voucher Code** | **Immersion in water at 80ºC** | **Preservative solution** | **Sample storage (days)** | **Processed larval mass (mg)** | **Quantification of total extracted DNA (ng/µl)** | **Solution absorbance at 260 nm**  **(A-260)** | **Solution absorbance at 280 nm**  **(A-280)** | **Spectrophotometric ratio**  **(260/280)** | **Obtaining mtDNA-COI amplicon** |
| --- | --- | --- | --- | --- | --- | --- | --- | --- | --- |
| CELEI-2025-1 | No | 99.3% ethanol | 7 | 19.5 | 271.1 | 5.421 | 2.597 | 2.1 | Yes |
| CELEI-2025-2 | No | 99.3% ethanol | 7 | 21.7 | 155.2 | 3.104 | 1.482 | 2.1 | Yes |
| CELEI- 2025-3 | No | 99.3% ethanol | 7 | 16.7 | 174.8 | 3.497 | 1.601 | 2.2 | Yes |
| CELEI-2025-4 | No | 70% ethanol | 7 | 20.3 | 74.3 | 1.486 | 0.714 | 2.1 | Yes |
| CELEI-2025-5 | No | 70% ethanol | 7 | 13.6 | 117.1 | 2.342 | 1.127 | 2.1 | Yes |
| CELEI-2025-6 | No | 70% ethanol | 7 | 16.9 | 103.4 | 2.068 | 0.977 | 2.1 | Yes |
| CELEI-2025-7 | No | 99.7% isopropyl alcohol | 7 | 12.7 | 38.4 | 0.768 | 0.414 | 1.8 | Yes |
| CELEI-2025-8 | No | 99.7% isopropyl alcohol | 7 | 13.2 | 51.4 | 1.028 | 0.545 | 1.9 | No |
| CELEI-2025-9 | No | 99.7% isopropyl alcohol | 7 | 21.2 | 58.8 | 1.175 | 0.705 | 1.7 | Yes |
| CELEI-2025-10 | No | Kahle’s solution | 7 | 22.1 | 183.7 | 3.675 | 1.751 | 2.1 | Yes |
| CELEI-2025-11 | No | Kahle’s solution | 7 | 20.3 | 154.9 | 3.098 | 1.521 | 2.0 | Yes |
| CELEI-2025-12 | No | Kahle’s solution | 7 | 25.7 | 144.3 | 2.886 | 1.315 | 2.2 | Yes |
| CELEI-2025-13 | Yes | 99.3% ethanol | 7 | 28.6 | 186.4 | 3.729 | 1.791 | 2.1 | Yes |
| CELEI-2025-14 | Yes | 99.3% ethanol | 7 | 18.3 | 73.2 | 1.464 | 0.673 | 2.2 | Yes |
| CELEI-2025-15 | Yes | 99.3% ethanol | 7 | 16.0 | 110.3 | 2.207 | 1.036 | 2.1 | Yes |

| **Voucher Code** | **Immersion in water at 80ºC** | **Preservative solution** | **Sample storage (days)** | **Processed larval mass (mg)** | **Quantification of total extracted DNA (ng/µl)** | **Solution absorbance at 260 nm**  **(A-260)** | **Solution absorbance at 280 nm**  **(A-280)** | **Spectrophotometric ratio**  **(260/280)** | **Obtaining mtDNA-COI amplicon** |
| --- | --- | --- | --- | --- | --- | --- | --- | --- | --- |
| CELEI-2025-16 | Yes | 70% ethanol | 7 | 23.5 | 242.5 | 4.849 | 2.286 | 2.1 | Yes |
| CELEI-2025-17 | Yes | 70% ethanol | 7 | 20.0 | 51.4 | 1.027 | 0.523 | 2.0 | Yes |
| CELEI-2025-18 | Yes | 70% ethanol | 7 | 14.1 | 50.9 | 1.019 | 0.557 | 1.8 | Yes |
| CELEI-2025-19 | Yes | 99.7% isopropyl alcohol | 7 | 15.9 | 43.1 | 0.861 | 0.404 | 2.1 | Yes |
| CELEI-2025-20 | Yes | 99.7% isopropyl alcohol | 7 | 16.6 | 55.4 | 1.108 | 0.523 | 2.1 | Yes |
| CELEI-2025-21 | Yes | 99.7% isopropyl alcohol | 7 | 23.1 | 102.3 | 2.047 | 0.991 | 2.1 | Yes |
| CELEI-2025-22 | Yes | Kahle’s solution | 7 | 19.9 | 48.6 | 0.972 | 0.464 | 2.1 | Yes |
| CELEI-2025-23 | Yes | Kahle’s solution | 7 | 26.5 | 46.2 | 0.924 | 0.447 | 2.1 | Yes |
| CELEI-2025-24 | Yes | Kahle’s solution | 7 | 31.9 | 96.7 | 1.933 | 0.951 | 2.0 | Yes |
| CELEI-2025-25 | No | 99.3% ethanol | 12 | 19.2 | 197.2 | 3.945 | 1.898 | 2.1 | No |
| CELEI-2025-26 | No | 99.3% ethanol | 12 | 20.4 | 146.6 | 2.931 | 1.367 | 2.1 | Yes |
| CELEI-2025-27 | No | 99.3% ethanol | 12 | 15.9 | 206.0 | 4.121 | 1.951 | 2.1 | Yes |
| CELEI-2025-28 | No | 70% ethanol | 12 | 12.3 | 41.5 | 0.829 | 0.404 | 2.0 | Yes |
| CELEI-2025-29 | No | 70% ethanol | 12 | 22.5 | 83.9 | 1.677 | 0.836 | 2.0 | Yes |
| CELEI-2025-30 | No | 70% ethanol | 12 | 23.8 | 43.1 | 0.862 | 0.421 | 2.0 | Yes |
| CELEI-2025-31 | No | 99.7% isopropyl alcohol | 12 | 8.1 | 32.9 | 0.658 | 0.387 | 1.7 | No |
| CELEI-2025-32 | No | 99.7% isopropyl alcohol | 12 | 13.1 | 41.5 | 0.830 | 0.401 | 2.1 | No |
| CELEI-2025-33 | No | 99.7% isopropyl alcohol | 12 | 13.3 | 47.7 | 0.954 | 0.504 | 1.9 | Yes |
| CELEI-2025-34 | No | Kahle’s solution | 12 | 21.7 | 154.8 | 3.095 | 1.503 | 2.1 | Yes |
| CELEI-2025-35 | No | Kahle’s solution | 12 | 25.5 | 176.6 | 3.532 | 1.734 | 2.0 | Yes |
| **Voucher Code** | **Immersion in water at 80ºC** | **Preservative solution** | **Sample storage (days)** | **Processed larval mass (mg)** | **Quantification of total extracted DNA (ng/µl)** | **Solution absorbance at 260 nm**  **(A-260)** | **Solution absorbance at 280 nm**  **(A-280)** | **Spectrophotometric ratio**  **(260/280)** | **Obtaining mtDNA-COI amplicon** |
| CELEI-2025-36 | No | Kahle’s solution | 12 | 21.5 | 246.1 | 4.922 | 2.347 | 2.1 | Yes |
| CELEI-2025-37 | Yes | 99.3% ethanol | 12 | 21.7 | 118.6 | 2.373 | 1.068 | 2.2 | Yes |
| CELEI-2025-38 | Yes | 99.3% ethanol | 12 | 28.5 | 619.4 | 12.388 | 6.268 | 2.0 | Yes |
| CELEI-2025-39 | Yes | 99.3% ethanol | 12 | 21.3 | 36.5 | 0.730 | 0.364 | 2.0 | Yes |
| CELEI-2025-40 | Yes | 70% ethanol | 12 | 24.0 | 81.9 | 1.638 | 0.787 | 2.1 | Yes |
| CELEI-2025-41 | Yes | 70% ethanol | 12 | 24.6 | 174.8 | 3.496 | 1.853 | 1.9 | Yes |
| CELEI- 2025-42 | Yes | 70% ethanol | 12 | 17.8 | 72.4 | 1.447 | 0.764 | 1.9 | Yes |
| CELEI-2025-43 | Yes | 99.7% isopropyl alcohol | 12 | 19.3 | 61.9 | 1.237 | 0.633 | 1.9 | Yes |
| CELEI-2025-44 | Yes | 99.7% isopropyl alcohol | 12 | 14.7 | 189.5 | 3.791 | 1.818 | 2.1 | Yes |
| CELEI-2025-45 | Yes | 99.7% isopropyl alcohol | 12 | 14.3 | 35.6 | 0.711 | 0.367 | 1.9 | Yes |
| CELEI-2025-46 | Yes | Kahle’s solution | 12 | 26.3 | 195.2 | 3.905 | 2.013 | 1.9 | No |
| CELEI-2025-47 | Yes | Kahle’s solution | 12 | 30.1 | 129.0 | 2.580 | 1.252 | 2.1 | No |
| CELEI-2025-48 | Yes | Kahle’s solution | 12 | 28.0 | 86.0 | 1.719 | 0.870 | 2.0 | No |
| CELEI-2025-49 | No | 99.3% ethanol | 18 | 20.0 | 282.4 | 5.648 | 2.598 | 2.2 | Yes |
| CELEI-2025-50 | No | 99.3% ethanol | 18 | 22.6 | 234.7 | 4.693 | 2.158 | 2.2 | Yes |
| CELEI-2025-51 | No | 99.3% ethanol | 18 | 15.4 | 500.4 | 10.008 | 4.552 | 2.2 | Yes |
| CELEI-2025-52 | No | 70% ethanol | 18 | 23.6 | 87.6 | 1.752 | 0.983 | 1.8 | Yes |
| CELEI-2025-53 | No | 70% etanol | 18 | 13.2 | 26.5 | 0.530 | 0.286 | 1.8 | No |
| CELEI-2025-54 | No | 70% etanol | 18 | 22.7 | 64.3 | 1.286 | 0.666 | 1.9 | Yes |
| CELEI-2025-55 | No | 99.7% isopropyl alcohol | 18 | 13.0 | 29.3 | 0.587 | 0.287 | 2.0 | No |
| **Voucher Code** | **Immersion in water at 80ºC** | **Preservative solution** | **Sample storage (days)** | **Processed larval mass (mg)** | **Quantification of total extracted DNA (ng/µl)** | **Solution absorbance at 260 nm**  **(A-260)** | **Solution absorbance at 280 nm**  **(A-280)** | **Spectrophotometric ratio**  **(260/280)** | **Obtaining mtDNA-COI amplicon** |
| CELEI-2025-56 | No | 99.7% isopropyl alcohol | 18 | 15.6 | 37.6 | 0.751 | 0.389 | 1.9 | Yes |
| CELEI-2025-57 | No | 99.7% isopropyl alcohol | 18 | 17.1 | 65.7 | 1.313 | 0.772 | 1.7 | Yes |
| CELEI- 2025-58 | No | Kahle’s solution | 18 | 37.4 | 166.0 | 3.320 | 1.611 | 2.1 | Yes |
| CELEI-2025-59 | No | Kahle’s solution | 18 | 19.3 | 62.7 | 1.254 | 0.596 | 2.1 | Yes |
| CELEI- 2025-60 | No | Kahle’s solution | 18 | 23.1 | 41.4 | 0.828 | 0.466 | 1.8 | Yes |
| CELEI-2025-61 | Yes | 99.3% ethanol | 18 | 24.6 | 82.9 | 1.657 | 0.782 | 2.1 | Yes |
| CELEI- 2025-62 | Yes | 99.3% ethanol | 18 | 30.3 | 71.7 | 1.434 | 0.675 | 2.1 | Yes |
| CELEI-2025-63 | Yes | 99.3% ethanol | 18 | 20.5 | 161.8 | 3.235 | 1.470 | 2.2 | Yes |
| CELEI- 2025-64 | Yes | 70% ethanol | 18 | 16.2 | 75.7 | 1.513 | 0.692 | 2.2 | Yes |
| CELEI-2025-65 | Yes | 70% ethanol | 18 | 20.1 | 116.2 | 2.324 | 1.052 | 2.2 | Yes |
| CELEI- 2025-66 | Yes | 70% ethanol | 18 | 29.0 | 45.9 | 0.918 | 0.478 | 1.9 | Yes |
| CELEI-2025-67 | Yes | 99.7% isopropyl alcohol | 18 | 14.0 | 100.3 | 2.005 | 0.910 | 2.2 | Yes |
| CELEI- 2025-68 | Yes | 99.7% isopropyl alcohol | 18 | 16.1 | 233.8 | 4.676 | 2.092 | 2.2 | Yes |
| CELEI-2025-69 | Yes | 99.7% isopropyl alcohol | 18 | 17.6 | 96.0 | 1.920 | 0.892 | 2.1 | Yes |
| CELEI- 2025-70 | Yes | Kahle’s solution | 18 | 27.6 | 60.8 | 1.216 | 0.607 | 2.0 | No |
| CELEI-2025-71 | Yes | Kahle’s solution | 18 | 21.8 | 34.5 | 0.690 | 0.330 | 2.1 | No |
| CELEI- 2025-72 | Yes | Kahle’s solution | 18 | 39.2 | 45.4 | 0.909 | 0.479 | 1.9 | No |
| CELEI-2025-73 | No | 99.3% ethanol | 28 | 11.8 | 61.5 | 1.231 | 0.577 | 2.1 | Yes |
| CELEI- 2025-74 | No | 99.3% ethanol | 28 | 19.2 | 142.9 | 2.859 | 1.380 | 2.1 | Yes |
| CELEI-2025-75 | No | 99.3% ethanol | 28 | 18.4 | 125.8 | 2.517 | 1.224 | 2.1 | Yes |
| **Voucher Code** | **Immersion in water at 80ºC** | **Preservative solution** | **Sample storage (days)** | **Processed larval mass (mg)** | **Quantification of total extracted DNA (ng/µl)** | **Solution absorbance at 260 nm**  **(A-260)** | **Solution absorbance at 280 nm**  **(A-280)** | **Spectrophotometric ratio**  **(260/280)** | **Obtaining mtDNA-COI amplicon** |
| CELEI-2025-76 | No | 70% ethanol | 28 | 22.0 | 65.3 | 1.306 | 0.635 | 2.1 | Yes |
| CELEI-2025-77 | No | 70% ethanol | 28 | 29.0 | 104.9 | 2.097 | 1.114 | 1.9 | Yes |
| CELEI- 2025-78 | No | 70% ethanol | 28 | 13.3 | 33.3 | 0.665 | 0.355 | 1.9 | No |
| CELEI-2025-79 | No | 99.7% isopropyl alcohol | 28 | 16.6 | 37.2 | 0.743 | 0.364 | 2.0 | Yes |
| CELEI- 2025-80 | No | 99.7% isopropyl alcohol | 28 | 13.7 | 50.6 | 1.011 | 0.569 | 1.8 | Yes |
| CELEI-2025-81 | No | 99.7% isopropyl alcohol | 28 | 14.0 | 113.0 | 2.259 | 1.233 | 1.8 | No |
| CELEI- 2025-82 | No | Kahle’s solution | 28 | 23.9 | 21.0 | 0.420 | 0.250 | 1.7 | No |
| CELEI-2025-83 | No | Kahle’s solution | 28 | 23.6 | 66.6 | 1.332 | 0.669 | 2.0 | Yes |
| CELEI- 2025-84 | No | Kahle’s solution | 28 | 24.8 | 52.0 | 1.041 | 0.627 | 1.7 | No |
| CELEI-2025-85 | Yes | 99.3% ethanol | 28 | 21.1 | 71.6 | 1.432 | 0.726 | 2.0 | Yes |
| CELEI- 2025-86 | Yes | 99.3% ethanol | 28 | 16.8 | 64.8 | 1.297 | 0.693 | 1.9 | Yes |
| CELEI-2025-87 | Yes | 99.3% ethanol | 28 | 19.0 | 68,2 | 1,365 | 0,710 | 1.9 | No |
| CELEI- 2025-88 | Yes | 70% ethanol | 28 | 22.8 | 67.7 | 1.354 | 0.698 | 1.9 | Yes |
| CELEI-2025-89 | Yes | 70% ethanol | 28 | 14.3 | 82.4 | 1.648 | 0.838 | 2.0 | Yes |
| CELEI- 2025-90 | Yes | 70% ethanol | 28 | 27.9 | 67.2 | 1.343 | 0.819 | 1.6 | Yes |
| CELEI-2025-91 | Yes | 99.7% isopropyl alcohol | 28 | 14.6 | 55.1 | 1.101 | 0.523 | 2.1 | Yes |
| CELEI- 2025-92 | Yes | 99.7% isopropyl alcohol | 28 | 14.0 | 109.0 | 2.179 | 1.046 | 2.1 | Yes |
| CELEI-2025-93 | Yes | 99.7% isopropyl alcohol | 28 | 16.4 | 79.1 | 1.583 | 0.808 | 2.0 | No |
| CELEI- 2025-94 | Yes | Kahle’s solution | 28 | 26.7 | 47.2 | 0.944 | 0.507 | 1.9 | Yes |
| CELEI-2025-95 | Yes | Kahle’s solution | 28 | 24.9 | 76.3 | 1.527 | 0.886 | 1.7 | No |
| **Voucher Code** | **Immersion in water at 80ºC** | **Preservative solution** | **Sample storage (days)** | **Processed larval mass (mg)** | **Quantification of total extracted DNA (ng/µl)** | **Solution absorbance at 260 nm**  **(A-260)** | **Solution absorbance at 280 nm**  **(A-280)** | **Spectrophotometric ratio**  **(260/280)** | **Obtaining mtDNA-COI amplicon** |
| CELEI-2025-96 | Yes | Kahle’s solution | 28 | 25.8 | 61.8 | 1.236 | 0.697 | 1.8 | No |
| CETdeA #21467 | No | 99.3% ethanol | 47 | 20.1 | 2.1 | N.A. | N.A. | N.A. | No |
| CETdeA #21468 | No | 99.3% ethanol | 47 | 20.1 | 1.5 | N.A. | N.A. | N.A. | No |
| CETdeA #21469 | No | 99.3% ethanol | 47 | 20.1 | 1.2 | N.A. | N.A. | N.A. | Yes |
| CETdeA #21470 | No | 99.3% ethanol | 47 | 20.1 | 1.2 | N.A. | N.A. | N.A. | Yes |
| CETdeA #21471 | No | 99.3% ethanol | 47 | 20.1 | 1.6 | N.A. | N.A. | N.A. | Yes |
| CETdeA #21472 | No | 99.3% ethanol | 47 | 20.1 | 2.2 | N.A. | N.A. | N.A. | No |
| CETdeA #21473 | No | 99.3% ethanol | 47 | 20.1 | 1.6 | N.A. | N.A. | N.A. | Yes |
| CETdeA #21474 | No | 99.3% ethanol | 47 | 20.1 | 2.1 | N.A. | N.A. | N.A. | Yes |
| CETdeA #21475 | No | 99.3% ethanol | 47 | 20.1 | 3.1 | N.A. | N.A. | N.A. | Yes |
| CETdeA #21476 | No | 99.3% ethanol | 47 | 20.1 | 2.7 | N.A. | N.A. | N.A. | No |
| CETdeA #21457 | No | 70% ethanol | 47 | 20.1 | 2.1 | N.A. | N.A. | N.A. | Yes |
| CETdeA #21458 | No | 70% ethanol | 47 | 20.1 | 1.4 | N.A. | N.A. | N.A. | Yes |
| CETdeA #21459 | No | 70% ethanol | 47 | 20.1 | 1.1 | N.A. | N.A. | N.A. | Yes |
| CETdeA #21460 | No | 70% ethanol | 47 | 20.1 | 1.2 | N.A. | N.A. | N.A. | Yes |
| CETdeA #21461 | No | 70% ethanol | 47 | 20.1 | 1.7 | N.A. | N.A. | N.A. | Yes |
| CETdeA #21462 | No | 70% ethanol | 47 | 20.1 | 1.4 | N.A. | N.A. | N.A. | No |
| CETdeA #21463 | No | 70% ethanol | 47 | 20.1 | 1.2 | N.A. | N.A. | N.A. | Yes |
| CETdeA #21464 | No | 70% ethanol | 47 | 20.1 | 1.7 | N.A. | N.A. | N.A. | No |
| CETdeA #21465 | No | 70% ethanol | 47 | 20.1 | 2.0 | N.A. | N.A. | N.A. | Yes |
| **Voucher Code** | **Immersion in water at 80ºC** | **Preservative solution** | **Sample storage (days)** | **Processed larval mass (mg)** | **Quantification of total extracted DNA (ng/µl)** | **Solution absorbance at 260 nm**  **(A-260)** | **Solution absorbance at 280 nm**  **(A-280)** | **Spectrophotometric ratio**  **(260/280)** | **Obtaining mtDNA-COI amplicon** |
| CETdeA #21466 | No | 70% ethanol | 47 | 20.1 | 1.2 | N.A. | N.A. | N.A. | No |
| CETdeA #21477 | No | 99.7% isopropyl alcohol | 47 | 20.1 | < 1.0 | N.A. | N.A. | N.A. | Yes |
| CETdeA #21478 | No | 99.7% isopropyl alcohol | 47 | 20.1 | 1.3 | N.A. | N.A. | N.A. | Yes |
| CETdeA #21479 | No | 99.7% isopropyl alcohol | 47 | 20.1 | 1.1 | N.A. | N.A. | N.A. | Yes |
| CETdeA #21480 | No | 99.7% isopropyl alcohol | 47 | 20.1 | 1.0 | N.A. | N.A. | N.A. | No |
| CETdeA #21481 | No | 99.7% isopropyl alcohol | 47 | 20.1 | < 1.0 | N.A. | N.A. | N.A. | Yes |
| CETdeA #21482 | No | 99.7% isopropyl alcohol | 47 | 20.1 | < 1.0 | N.A. | N.A. | N.A. | Yes |
| CETdeA #21483 | No | 99.7% isopropyl alcohol | 47 | 20.1 | < 1.0 | N.A. | N.A. | N.A. | No |
| CETdeA #21484 | No | 99.7% isopropyl alcohol | 47 | 20.1 | 1.0 | N.A. | N.A. | N.A. | No |
| CETdeA #21485 | No | 99.7% isopropyl alcohol | 47 | 20.1 | < 1.0 | N.A. | N.A. | N.A. | No |
| CETdeA #21486 | No | 99.7% isopropyl alcohol | 47 | 20.1 | < 1.0 | N.A. | N.A. | N.A. | No |
| CETdeA #21487 | No | Kahle’s solution | 47 | 20.1 | 2.0 | N.A. | N.A. | N.A. | No |
| CETdeA #21488 | No | Kahle’s solution | 47 | 20.1 | < 1.0 | N.A. | N.A. | N.A. | No |
| CETdeA #21489 | No | Kahle’s solution | 47 | 20.1 | 2.2 | N.A. | N.A. | N.A. | No |
| CETdeA #21490 | No | Kahle’s solution | 47 | 20.1 | 1.9 | N.A. | N.A. | N.A. | No |
| CETdeA #21491 | No | Kahle’s solution | 47 | 20.1 | 1.6 | N.A. | N.A. | N.A. | No |
| CETdeA #21492 | No | Kahle’s solution | 47 | 20.1 | 1.8 | N.A. | N.A. | N.A. | No |
| CETdeA #21493 | No | Kahle’s solution | 47 | 20.1 | 1.7 | N.A. | N.A. | N.A. | No |
| CETdeA #21494 | No | Kahle’s solution | 47 | 20.1 | 1.9 | N.A. | N.A. | N.A. | No |
| CETdeA #21495 | No | Kahle’s solution | 47 | 20.1 | 1.9 | N.A. | N.A. | N.A. | No |
| **Voucher Code** | **Immersion in water at 80ºC** | **Preservative solution** | **Sample storage (days)** | **Processed larval mass (mg)** | **Quantification of total extracted DNA (ng/µl)** | **Solution absorbance at 260 nm**  **(A-260)** | **Solution absorbance at 280 nm**  **(A-280)** | **Spectrophotometric ratio**  **(260/280)** | **Obtaining mtDNA-COI amplicon** |
| CETdeA #21496 | No | Kahle’s solution | 47 | 20.1 | 1.6 | N.A. | N.A. | N.A. | No |
| CETdeA #21427 | Yes | 99.3% ethanol | 47 | 20.1 | 3.7 | N.A. | N.A. | N.A. | No |
| CETdeA #21428 | Yes | 99.3% ethanol | 47 | 20.1 | 1.1 | N.A. | N.A. | N.A. | No |
| CETdeA #21429 | Yes | 99.3% ethanol | 47 | 20.1 | 4.5 | N.A. | N.A. | N.A. | Yes |
| CETdeA #21430 | Yes | 99.3% ethanol | 47 | 20.1 | < 1.0 | N.A. | N.A. | N.A. | No |
| CETdeA #21431 | Yes | 99.3% ethanol | 47 | 20.1 | 3.0 | N.A. | N.A. | N.A. | No |
| CETdeA #21432 | Yes | 99.3% ethanol | 47 | 20.1 | 3.7 | N.A. | N.A. | N.A. | No |
| CETdeA #21433 | Yes | 99.3% ethanol | 47 | 20.1 | < 1.0 | N.A. | N.A. | N.A. | No |
| CETdeA #21434 | Yes | 99.3% ethanol | 47 | 20.1 | 8.1 | N.A. | N.A. | N.A. | Yes |
| CETdeA #21435 | Yes | 99.3% ethanol | 47 | 20.1 | 1.5 | N.A. | N.A. | N.A. | No |
| CETdeA #21436 | Yes | 99.3% ethanol | 47 | 20.1 | 6.0 | N.A. | N.A. | N.A. | No |
| CETdeA #21417 | Yes | 70% ethanol | 47 | 20.1 | 11.5 | N.A. | N.A. | N.A. | No |
| CETdeA #21418 | Yes | 70% ethanol | 47 | 20.1 | 13.0 | N.A. | N.A. | N.A. | No |
| CETdeA #21419 | Yes | 70% ethanol | 47 | 20.1 | 21.7 | N.A. | N.A. | N.A. | No |
| CETdeA #21420 | Yes | 70% ethanol | 47 | 20.1 | 30.1 | N.A. | N.A. | N.A. | Yes |
| CETdeA #21421 | Yes | 70% ethanol | 47 | 20.1 | 31.2 | N.A. | N.A. | N.A. | Yes |
| CETdeA #21422 | Yes | 70% ethanol | 47 | 20.1 | 13.1 | N.A. | N.A. | N.A. | Yes |
| CETdeA #21423 | Yes | 70% ethanol | 47 | 20.1 | 24.9 | N.A. | N.A. | N.A. | Yes |
| CETdeA #21424 | Yes | 70% ethanol | 47 | 20.1 | 11.8 | N.A. | N.A. | N.A. | No |
| CETdeA #21425 | Yes | 70% ethanol | 47 | 20.1 | 15.2 | N.A. | N.A. | N.A. | Yes |
| **Voucher Code** | **Immersion in water at 80ºC** | **Preservative solution** | **Sample storage (days)** | **Processed larval mass (mg)** | **Quantification of total extracted DNA (ng/µl)** | **Solution absorbance at 260 nm**  **(A-260)** | **Solution absorbance at 280 nm**  **(A-280)** | **Spectrophotometric ratio**  **(260/280)** | **Obtaining mtDNA-COI amplicon** |
| CETdeA #21426 | Yes | 70% ethanol | 47 | 20.1 | 21.6 | N.A. | N.A. | N.A. | Yes |
| CETdeA #21437 | Yes | 99.7% isopropyl alcohol | 47 | 20.1 | 7.0 | N.A. | N.A. | N.A. | Yes |
| CETdeA #21438 | Yes | 99.7% isopropyl alcohol | 47 | 20.1 | < 1.0 | N.A. | N.A. | N.A. | No |
| CETdeA #21439 | Yes | 99.7% isopropyl alcohol | 47 | 20.1 | 10.1 | N.A. | N.A. | N.A. | Yes |
| CETdeA #21440 | Yes | 99.7% isopropyl alcohol | 47 | 20.1 | 9.3 | N.A. | N.A. | N.A. | Yes |
| CETdeA #21441 | Yes | 99.7% isopropyl alcohol | 47 | 20.1 | 17.6 | N.A. | N.A. | N.A. | Yes |
| CETdeA #21442 | Yes | 99.7% isopropyl alcohol | 47 | 20.1 | 9.3 | N.A. | N.A. | N.A. | Yes |
| CETdeA #21443 | Yes | 99.7% isopropyl alcohol | 47 | 20.1 | < 1.0 | N.A. | N.A. | N.A. | No |
| CETdeA #21444 | Yes | 99.7% isopropyl alcohol | 47 | 20.1 | < 1.0 | N.A. | N.A. | N.A. | No |
| CETdeA #21445 | Yes | 99.7% isopropyl alcohol | 47 | 20.1 | < 1.0 | N.A. | N.A. | N.A. | No |
| CETdeA #21446 | Yes | 99.7% isopropyl alcohol | 47 | 20.1 | < 1.0 | N.A. | N.A. | N.A. | No |
| CETdeA #21447 | Yes | Kahle’s solution | 47 | 20.1 | < 1.0 | N.A. | N.A. | N.A. | No |
| CETdeA #21448 | Yes | Kahle’s solution | 47 | 20.1 | < 1.0 | N.A. | N.A. | N.A. | No |
| CETdeA #21449 | Yes | Kahle’s solution | 47 | 20.1 | < 1.0 | N.A. | N.A. | N.A. | No |
| CETdeA #21450 | Yes | Kahle’s solution | 47 | 20.1 | 1.2 | N.A. | N.A. | N.A. | No |
| CETdeA #21451 | Yes | Kahle’s solution | 47 | 20.1 | < 1.0 | N.A. | N.A. | N.A. | No |
| CETdeA #21452 | Yes | Kahle’s solution | 47 | 20.1 | < 1.0 | N.A. | N.A. | N.A. | No |
| CETdeA #21453 | Yes | Kahle’s solution | 47 | 20.1 | 4.0 | N.A. | N.A. | N.A. | No |
| CETdeA #21454 | Yes | Kahle’s solution | 47 | 20.1 | 4.8 | N.A. | N.A. | N.A. | No |
| CETdeA #21455 | Yes | Kahle’s solution | 47 | 20.1 | 2.3 | N.A. | N.A. | N.A. | No |
| **Voucher Code** | **Immersion in water at 80ºC** | **Preservative solution** | **Sample storage (days)** | **Processed larval mass (mg)** | **Quantification of total extracted DNA (ng/µl)** | **Solution absorbance at 260 nm**  **(A-260)** | **Solution absorbance at 280 nm**  **(A-280)** | **Spectrophotometric ratio**  **(260/280)** | **Obtaining mtDNA-COI amplicon** |
| CETdeA #21456 | Yes | Kahle’s solution | 47 | 20.1 | 1.5 | N.A. | N.A. | N.A. | No |

**Note**: N.A. = parameter not analyzed in the Qubit™ since this system is more sensitive for DNA quantification. CELEI and CETdeA represent the acronyms of the entomological collections where the vouchers were deposited.
