# Supplementary figures and images for: DNA Recovery from Forensically Relevant Blow Fly Larvae (Insecta, Diptera, Calliphoridae) Kept in Different Preservative Solutions
Source: Neotrop Entomol. 2026 Feb 20;55(1):14. doi: 10.1007/s13744-026-01366-x (PMC12920309; doi:10.1007/s13744-026-01366-x)

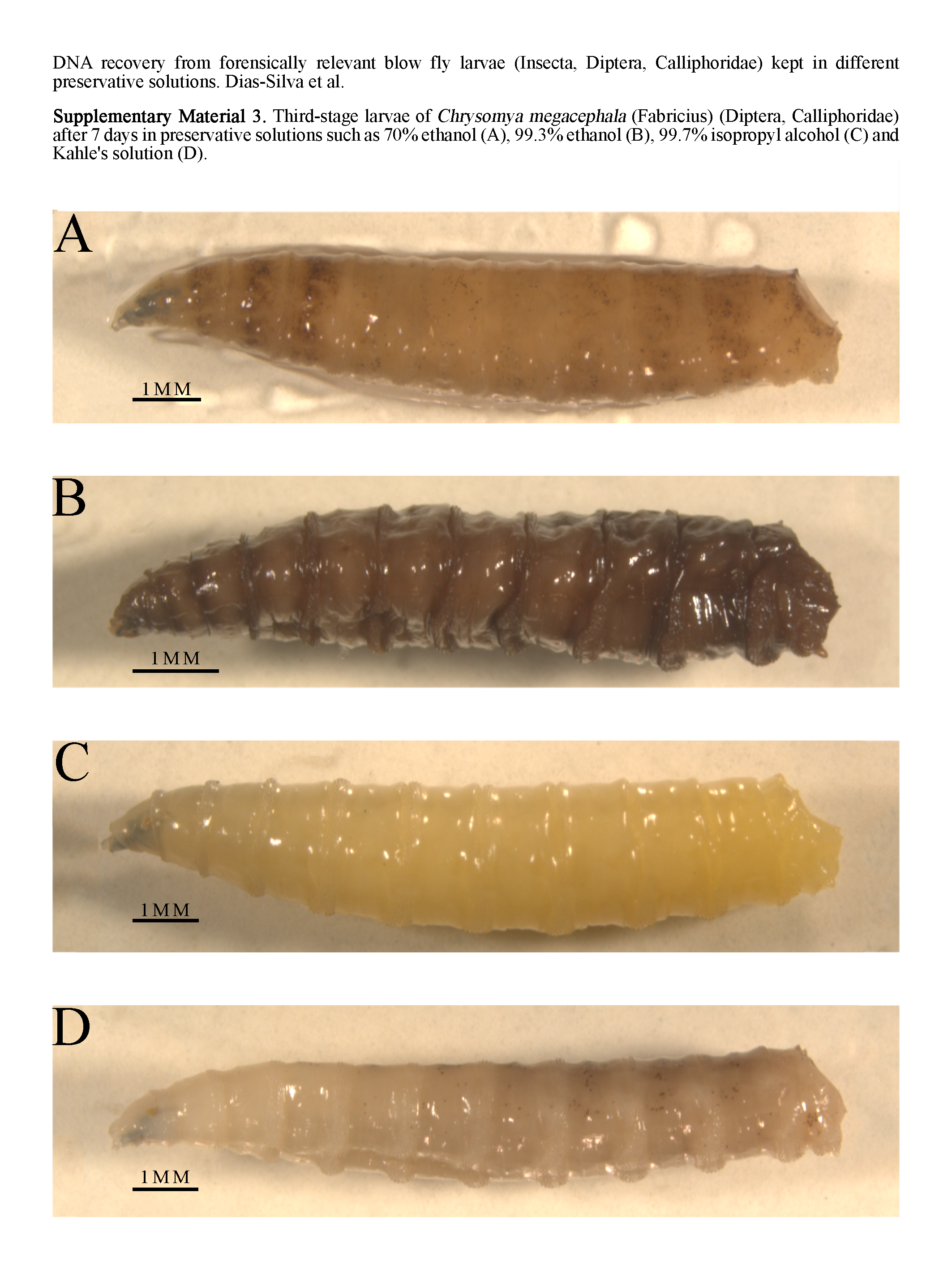

Supplement: Supplementary file 3 — (PNG 3.47 MB) [file 13744_2026_1366_Fig4_ESM.png]

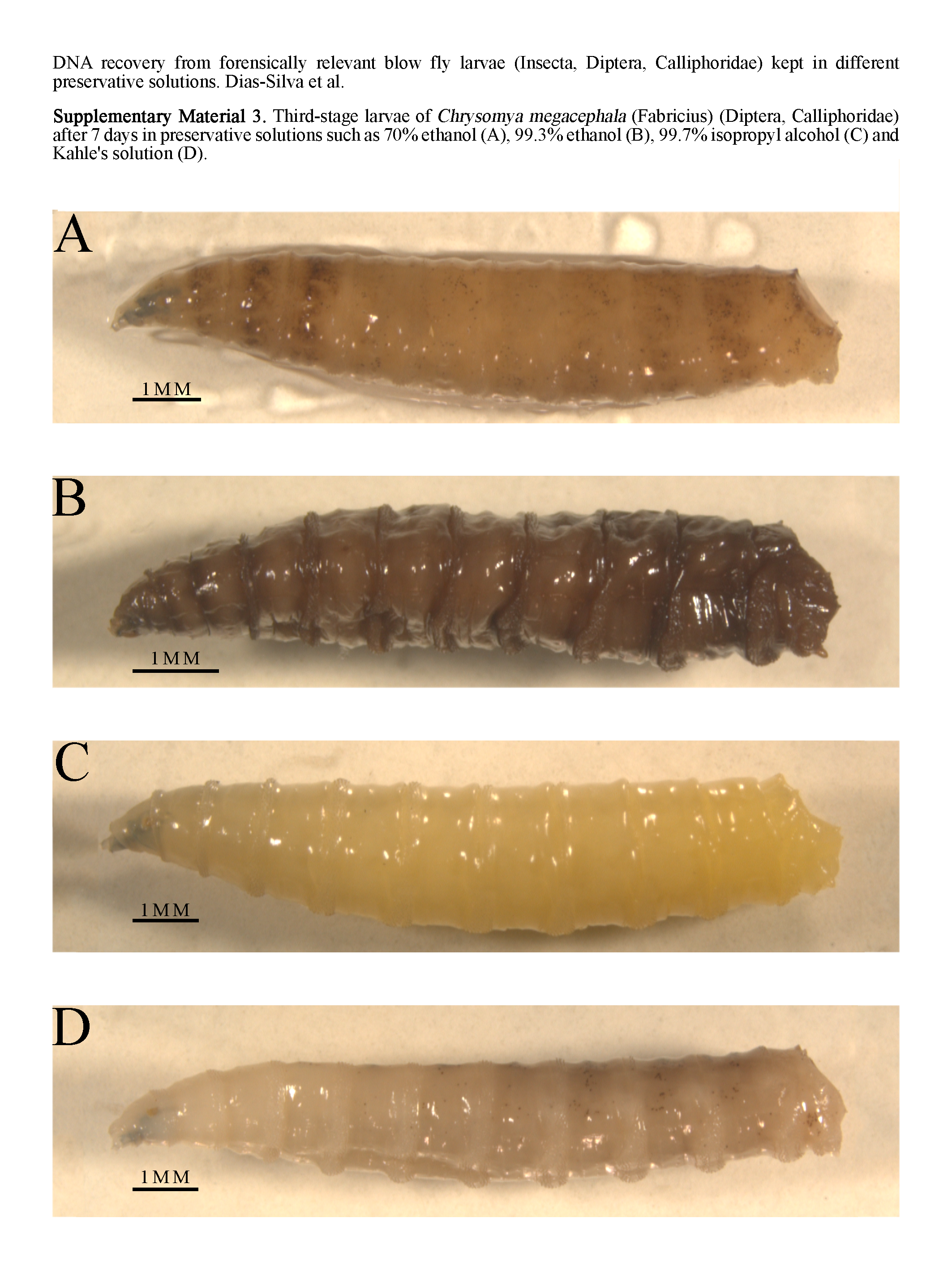

Supplement: Supplementary file 4 — High Resolution Image (34.6 MB) [file 13744_2026_1366_MOESM3_ESM.tif]
